# Neurological involvement in hematopoietic stem cell transplantation-associated thrombotic microangiopathy

**DOI:** 10.1007/s00277-024-05798-6

**Published:** 2024-05-20

**Authors:** Wanying Liu, Xiaojian Zhu, Yi Xiao

**Affiliations:** Department of Hematology, Tongji Hospital, Tongji Medical College, Huazhong University of Science and Technology, Wuhan, Hubei China

**Keywords:** Endothelial injury, Neurological involvement, Hematopoietic stem cell transplantation, Thrombotic microangiopathy

## Abstract

Transplantation-associated thrombotic microangiopathy (TA-TMA) is a well-recognized serious complication of hematopoietic stem cell transplantation (HSCT). The understanding of TA-TMA pathophysiology has expanded in recent years. Dysregulation of the complement system is thought to cause endothelial injury and, consequently, microvascular thrombosis and tissue damage. TA-TMA can affect multiple organs, and each organ exhibits specific features of injury. Central nervous system (CNS) manifestations of TA-TMA include posterior reversible encephalopathy syndrome, seizures, and encephalopathy. The development of neurological dysfunction is associated with a significantly lower overall survival in patients with TA-TMA. However, there are currently no established histopathological or radiological criteria for the diagnosis of CNS TMA. Patients who receive total body irradiation (TBI), calcineurin inhibitors (CNI), and severe acute and chronic graft-versus-host disease (GVHD) are at a high risk of experiencing neurological complications related to TA-TMA and should be considered for directed TA-TMA therapy. However, the incidence and clinical manifestations of TA-TMA neurotoxicity remain unclear. Studies specifically examining the involvement of CNS in TMA syndromes are limited. In this review, we discuss clinical manifestations and imaging abnormalities in patients with nervous system involvement in TA-TMA. We summarize the mechanisms underlying TA-TMA and its neurological complications, including endothelial injury, evidence of complement activation, and treatment options for TA-TMA.

## Introduction

Thrombotic microangiopathy (TMA) causes global endothelial damage and multiorgan injury. Transplantation-associated thrombotic microangiopathy (TA-TMA) is a potentially fatal complication following hematopoietic stem cell transplantation (HSCT).

TMAs can be classified as primary TMAs, including atypical hemolytic uremic syndrome (aHUS) and thrombotic thrombocytopenic purpura (TTP), and secondary TMAs, which are associated with transplantation, pregnancy, malignancies, autoimmune diseases, drugs, and malignant hypertension. TA-TMA belongs to the family of thrombotic microangiopathies and, to some degree, shares similarities in terms of dysregulated complement activation to endothelial damage with other TMAs, especially aHUS. Recent studies have indicated a link between TA-TMA and systemic vascular endothelial injury [1, 2]. Endothelial injury is a common underlying cause of different TMA, leading to the microvasculature thrombosis observed in these conditions. The kidneys, intestine, stomach, brain, heart, lungs, liver, gallbladder, urinary bladder, ureter, oral cavity, pharynx, and esophagus can be affected in TA-TMA, with the kidney being the most frequently affected organ [3–11].

More than one cause or precipitating factor underlying TMAs is particularly common among patients with transplants. In a large-scale retrospective study involving 564 consecutive patients, evaluating the high frequency of multiple triggers or causes in patients with TMAs, published by Guillaume et al., “multiple hits” were found to be necessary to trigger the disease, especially in secondary TMAs [1]. Notably, multiple causative factors are known to contribute to the evolution of the “final common pathway” of endothelial injury in TA-TMA. Several risk factors published in retrospective studies have been associated with the development of TA-TMA after allogeneic HSCT; among these, the most notable is the endothelial injury caused by the transplantation and related infectious complications. HSCT conditioning agents including busulfan, fludarabine, platinum-based chemotherapy, and total body irradiation may increase the risk of TA-TMA [12]. Other medications commonly reported to be associated with TA-TMA include those used for graft-versus-host disease (GVHD) prophylaxis, especially the calcineurin inhibitors tacrolimus and cyclosporine, and the mammalian target of rapamycin (mTOR) inhibitor sirolimus [13]. Similarly, viral infections are often considered to be a “trigger” of TA-TMA, as patients showing signs of small vessel injury can also have concomitant infections caused by viruses, including cytomegalovirus, adenovirus, parvovirus B19, HHV-6, and BK virus [14–19]. Furthermore, elevated levels of circulating cytokines, including IL-8, IL-12, and thrombomodulin, render endothelial cells more susceptible to apoptosis and cell lysis during TA-TMA [20–22]. In this review, we summarize the underlying mechanism of TA-TMA and its neurological complications, including endothelial injury, complement activation, and treatment options for TA-TMA.

### Endothelial damage and dysfunction in TA-TMA

**Fig. 1:**
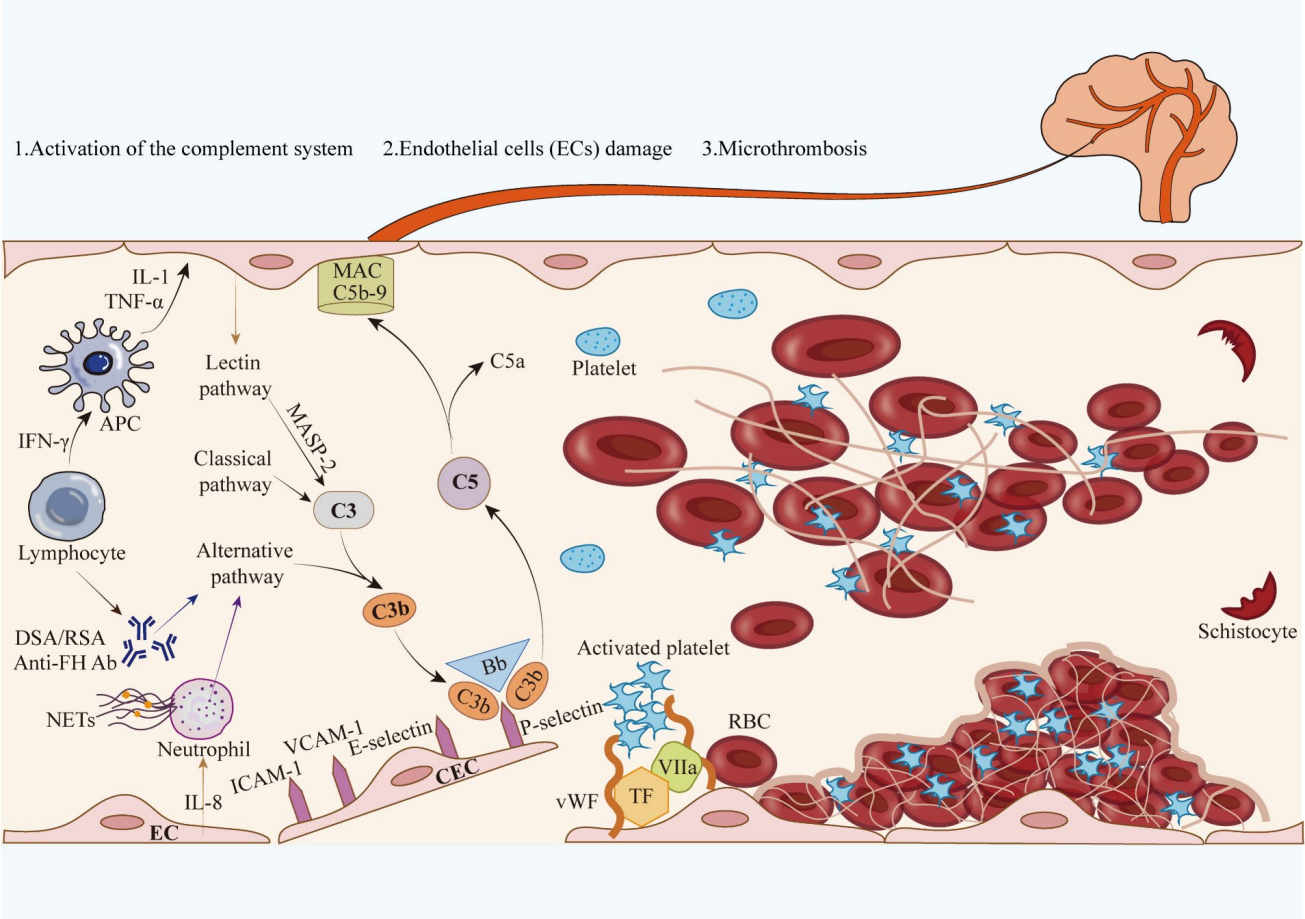
The illustration is demonstrates complement-mediated endothelial injury and microthrombi formation in transplant-associated thrombotic microangiopathy (TA-TMA). Activated lymphocytes produce IFN-γ, activating APCs. Subsequently, APCs produce TNF-α and IL-1, which damage vascular endothelial cells. The damaged ECs releases IL-8, causing neutrophil activation and release of neutrophil extracellular traps (NETs) leading to complement alternative activation. Activated lymphocytes also produce DSA/RSA and Anti-FH Ab, which similarly activate the complement alternative pathway. All three pathways converge to mediate cleavage of C3, initiating the terminal pathway to form the C5b-9 membrane attack complex and assembly of the MAC. Complement-mediated endothelial injury leads to the release of proinflammatory cytokines such as ICAM-1 and VCAM-1, as well as procoagulant factors like vWF, VIIa and TF. Additionally, there is an increase in the expression of adhesion molecules on the cell surface. All these phenomena promote further endothelial injury and leads to platelet aggregation and the initiation and propagation of the complement cascade. The erythrocytes are sheared and destroyed by platelet-rich microthrombi, leading to schistocyte production and microangiopathic hemolytic anemia. GVHD, graft-versus-host disease; EC, endothelial cell; CEC, circulating endothelial cell; APC, antigen-presenting cell; RBC, red blood cell; ICAM-1, intercellular adhesion molecule 1; VCAM-1, vascular adhesion molecule 1; vWF, von Willebrand factor; IFN, interferon; TNF, tumour necrosis factor; IL-1, interleukin 1; IL-8, interleukin 8; MAC,membrane attack complex; MASP-2, mannose-binding protein-associated serine protease-2; NETs, neutrophil extracellular traps; DSA, donor-specific antibody; RSA, recipient-specific antibody; Ab,antibody; VIIa, Factor VIIa; TF, tissue factor

Normally, the endothelium plays a role in maintaining homeostasis, including the regulation of coagulation, vascular tone, permeability and inflammatory processes. When activated, endothelial cells (ECs) acquire cellular features that may lead to phenotypic changes that induce pro-coagulant, pro-inflammatory and pro-apoptotic mediators. These mediators lead to EC dysfunction and damage, which may result in endothelial integrity disruption and multi-organ damage. EC dysfunction in the CNS damages cerebrovascular integrity, which manifests as multifocal hemorrhage. Cerebral hemorrhage occurs in different parts of the brain may cause different clinical manifestations. These mediators lead to EC dysfunction and damage, which may result in endothelial integrity disruption and multi-organ damage. EC dysfunction in the CNS damages cerebrovascular integrity, which manifests as multifocal hemorrhage. Cerebral hemorrhage occurs in different parts of the brain and may cause different clinical manifestations. In the context of HSCT, TMA occurs due to endothelial injury, which triggers the clotting cascade, leading to microangiopathic hemolytic anemia and platelet consumption. This cascade, in turn, results in thrombosis and fibrin deposition, ultimately inflicting widespread tissue injury. In TA-TMA, endothelial injury is multifactorial. The following three-hit hypothesis has been proposed and is widely accepted: hit 1: risk factors before HSCT, including older age, female sex, and secondary transplantation, may lead to pre-existing endothelial injury; hit 2: HSCT-related risk factors, including HLA mismatch, unrelated donor, total body radiation, myeloablative conditioning regimens, and calcineurin inhibitors (CNIs), may further injure endothelial cells; hit 3: risk factors after HSCT, including GVHD and infections, eventually lead to endothelial cell destruction [23]. Complement-mediated endothelial injury, intravascular platelet aggregation, microthrombi formation, narrowing of the vessel lumen, and mechanical damage lead to TA-TMA, cerebral vascular injury, and brain damage. The erythrocytes are sheared and destroyed by platelet-rich microthrombi, leading to schistocyte production and microangiopathic hemolytic anemia (Fig. 1). A procoagulant state in the endothelium results in direct endothelial damage. The generation of endothelial microparticles following EC activation and apoptosis may contribute to TA-TMA [24]. Circulating endothelial cells (CECs) serve as both a marker and mechanism of endothelial cell dysfunction because of their thrombotic and inflammatory properties [25]. CECs are markers of endothelial dysfunction and are used to monitor vascular damage in various situations, such as hematopoietic stem cell transplantation, acute myocardial infarction, and cardiovascular risk assessment [26–28]. Almici et al. analyzed CEC with the CellSearch system in 90 allogeneic hematopoietic stem cell transplantation (allo-HSCT) patients and concluded that CEC changes present a suitable biomarker to monitor endothelial damage in patients undergoing allo-HSCT [29]. Furthermore, CEC may be associated with a wide spectrum of endothelial complications during allo-HSCT, including GVHD, TMA, sinusoidal obstruction syndrome, capillary leak syndrome, and pulmonary arterial hypertension [28, 30]. Erdbruegger et al. studied CEC as markers of small blood vessel injury in 15 patients (five after HSCT) at different periods after TMA diagnosis, and reported that therapeutic plasma exchange (TPE) decreased the levels of CEC in patients who achieved a good clinical outcome [31]. Elevated levels of vWF have been observed along with other plasma markers of EC injury and inflammation, including thrombomodulin, plasminogen activator inhibitor-1, ICAM-1, VCAM-1, E-selectin, IL-1, TNFα, IFN-γ and IL-8 [17, 32]. In an autopsy study, Siami et al. concluded that endothelial injury may result from circulating cytokines, low levels of VEGF, activation of the coagulation pathway, or direct endothelial damage from cytotoxic donor T cells [10].

### Involvement of complement system

TMAs may result from the interaction of complement activation, genetic susceptibility, and environmental factors [33]. One of the best examples of such a mechanism underlies TA-TMA. The three complement-activated pathways converge at a common pathway that leads to the formation of strong inflammatory mediators, such as C3a and C5a, and the production of the C5b-9 membrane attack complex (MAC), which lyses the target cells [34, 35]. The role of the complement system has been well-established in the pathogenesis of other TMAs, most notably aHUS where defects in one or more regulatory proteins of the alternative pathway of the complement system allow for the uninhibited formation of C3 convertase C3bBb on the surface of endothelial cells. The formation of additional molecules of C3a, the activated form of C3, by this convertase on the cell surface leads to the formation of C5 convertase C3bBb•C3b, ultimately promoting the formation of the lytic complex C5b-9 and potent anaphylatoxin C5a; this leads to injury to endothelial cells and ultimately results in all the characteristic clinical findings of TMA [36].

Among the mechanisms responsible for complement activation in TA-TMA, structural and point alterations in a cluster of complement regulatory genes (*CFH, CFB, CFI, C3, CD46/MCP, DGKE, THBD,* and *CFHR*) are the most widely accepted [37, 38]. Genetic alterations in patients with TA-TMA were confirmed in a large prospective investigation by Jodele et al. In this study, pre-transplant genomic DNA was analyzed for 17 genes involved in the regulation of complement pathways in 77 pediatric patients undergoing HSCT (of whom 34 developed TA-TMA). Gene variants were detected in 65% of patients with TA-TMA (regardless of race) compared to 9% of patients without TMA [39]. Gavriilaki et al. confirmed these findings in 40 adult patients with TA-TMA, showing a significantly higher frequency of pathogenic and rare variants [40]. Endothelial injury directly activates the lectin pathway of the complement system, which subsequently activates the terminal lytic pathway gated by C5 [41]. Mannan-binding lectin-associated serine protease-2 (MASP-2) is an effector enzyme of the lectin pathway and activator of the coagulation cascade [42–44]. MASP-2 levels are elevated following HSCT in patients with TA-TMA [45]. Inhibition of MASP-2 may provide therapeutic benefits for TA-TMA and potentially other endothelial injury syndromes, including veno-occlusive disease/sinusoidal obstruction syndrome (VOD/SOS)and GVHD. Complement activation and endothelial injury are associated with TA-TMA [37]. Hale et al. reported normal C3, C4, and total complement activity (CH50) values in 11 children with HSCT-TMA [46]. A prospective study showed that upon HSCT-TMA diagnosis, patients with elevated levels of soluble terminal complement complex sC5b-9 had a 1-year survival rate of < 20%; by comparison, patients with normal serum C5b-9 levels had a survival rate of 100% [47]. In a study comparing patients with and without HSCT-TMA, Qi et al. showed that the levels of plasma C3b, soluble sC5b-9, and CH50 were significantly elevated in the former group (P < 0.001) [48].

## Multi-system endothelial injury

TA-TMA involving organs and histologic findings are summarized in Table 1. The kidney appears to be the most commonly affected organ by small-vessel injury associated with HSCT. Histopathological findings associated with TA-TMA include microthrombi in the glomeruli and characteristic patterns of C4d deposition in the renal arterioles [49]. Pulmonary arteriolar involvement in TA-TMA has been documented in several retrospective cohort studies following HSCT. Microangiopathic changes in pulmonary arterioles include denuded and injured endothelium, microthrombosis, and schistocyte extravasation into the lung interstitium [50–52]. TMA may involve the intestinal vasculature and can present with bleeding and ischemic colitis [53]. Polyserositis is a common but often missed feature of TA-TMA that results from a generalized vascular injury. It often presents as refractory pericardial effusion, pleural effusion, and ascites without generalized edema [54–56]. There is a growing body of evidence outlining the effects of TA-TMA on the small vasculature of the brain after HSCT. A study has revealed the classic vascular injury patterns of TMA in the brains of HSCT recipients, which confirmed the presence of CNS TA-TMA in this patient population. Neurological deficits have been reported in approximately six pediatric patients with TA-TMA [6].

**Table 1:** TA-TMA involving organs and histologic findings

TA-TMA involving organs	Involvement vessels	Histologic findings
Kidney	The glomerular capillaries; Vascular pole; Arterioles	Thickened capillary walls; Fragmented erythrocytes; Occluded vascular lumens; Endothelial separation with swelling; Fibrin deposition; Intraluminal microthrombi
Stomach/Intestine	Submucosal arterioles	Perivascular mucosal hemorrhage; Endothelial cell swelling; Intraluminal schistocytes separation; Intraluminal fibrin; Intraluminal microthrombi; Loss of glands; Mucosal denudation
Brain	CNS vasculature	Increased vascular wall-to-lumen ratios, Vascular basement membrane splitting ;Vascular wall reduplication, Perivascular hemosiderin deposition; Perivascular hemorrhage; Microinfarcts; Ischemic reperfusion injury and repair
Heart	Arterioles; Capillaries	Intimal thickening; arterioles narrowing; Artery thrombosis
Lung	Pulmonary small and medium-size arteries	Thickened arteries walls; Intraluminal microthrombi; Intimal arteritis; Red cell fragments; Perivascular lymphocytic infiltrate
Liver	Small arterioles	
Gallbladder	Small arteries	Erosion; Ulcers
Urinary bladder/Ureter	Small arteries in the mucosa or muscularis propria	
Oral cavity/Pharynx	Small arterioles	
Esophagus	Small arterioles	

## Potential mechanisms and pathology of TA-TMA with CNS involvement

The mechanisms of neurological manifestations of TA-TMA are not well understood, and further research is urgently warranted. Drawing on parallels from the endothelial injury mechanism observed in TMA (COVID-19 and CAR-T therapy-induced) in the nervous system, we can speculate that the mechanism of allo-HSCT-induced CNS TMA may also involve increased blood–brain barrier permeability owing to endothelial cell activation and damage [57–59]. A unifying hypothesis for CNS involvement in TMA syndrome is that endothelial dysfunction is related to the inflammatory response. Although the CNS vasculature can certainly be affected by TA-TMA, the most common TA-TMA-related CNS injury is likely due to acute uncontrolled TMA-associated hypertension, including PRES, which may result in CNS bleeding. Although brain biopsies are rarely performed, a detailed knowledge of TMA-mediated vascular injury patterns and the most common locations of these insults will improve our understanding of the clinical manifestations of CNS TMA. Neuroimaging reveals signal abnormalities in the posterior portions of the brain, including the brainstem, cerebellum, and basal ganglia, and symptoms are often preceded by significant hypertension [60]. Sabulski et al. described the widespread histological evidence of CNS vascular injury and subsequent tissue damage in patients with TA-TMA [6]. The histological features of TA-TMA within the CNS encompass several key elements, including global white matter loss, edema (global and perivascular), macrophage infiltration, demyelination, hydrocephalus ex vacuo, and gliosis. TA-TMA vascular-specific histological findings include increased vascular wall-to-lumen ratio, vascular basement membrane splitting, vascular wall reduplication, perivascular hemosiderin deposition, perivascular hemorrhage, microinfarcts, and ischemic reperfusion injury and repair.

## Neurologic symptoms and imaging findings in patients with TA-TMA

**Fig. 2:**
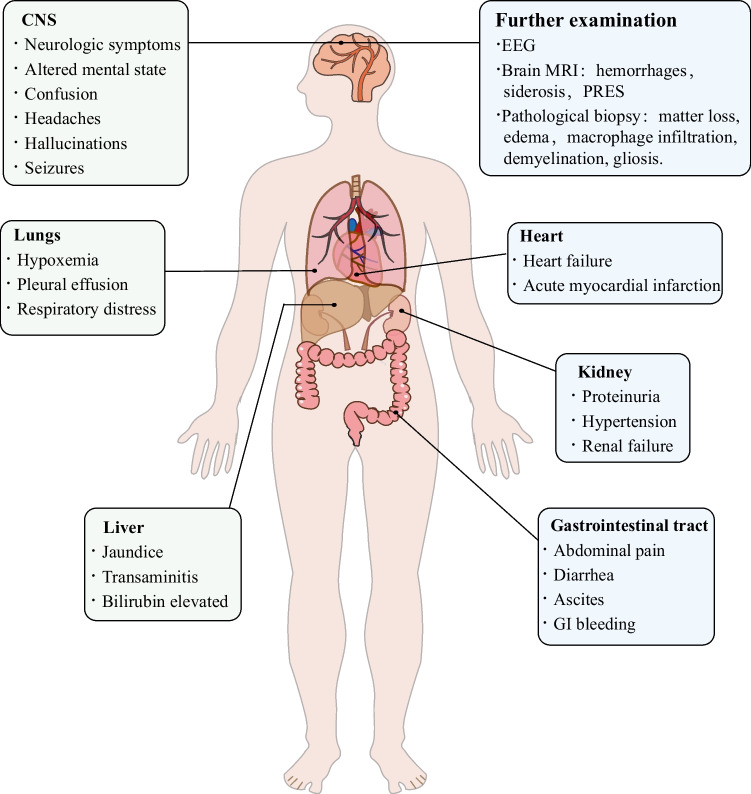
Central nervous system symptoms and other organ complications associated with TA-TMA after HSCT

Figure 2 shows central nervous system symptoms and other organ complications associated with TA-TMA. Neurological manifestations include altered mental state, memory loss, confusion, headaches, hallucinations, and seizures [61]. The development of neurological dysfunction is associated with significantly lower overall survival in patients with TA-TMA [62]. A separate study involving 221 children and adults noted that patients with TA-TMA were more likely to have neurological symptoms after HCT than those without TA-TMA (40% vs. 24%) [63]. In their risk stratification analysis of 130 HSCT recipients diagnosed with TA-TMA, Jodele et al. found that seizures and mental status changes associated with increased mortality risk were significant factors only in the univariate analysis; however this observation was not supported in the multivariate analysis [64]. No association was found between the presence of neurological manifestations (or specifically of encephalopathy or seizures) or neuroimaging abnormalities and poor outcomes at the last follow-up [65]. Brain imaging findings related to TMA include hemorrhages of various sizes, siderosis, and posterior reversible encephalopathy syndrome (PRES).

## Diagnosis and differential diagnosis of TA-TMA with CNS involvement

### Diagnostic and prognostic criteria

Currently, clinical diagnosis of TA-TMA is made using the criteria of Jodele et al [66]. Patients met ≥ 4 of the following 7 features at two different time points within 14 days: (1) lactate dehydrogenase above normal for age; (2) schistocytes on blood smear; (3) de novo thrombocytopenia or increased transfusion requirements; (4) de novo anemia or increased transfusion requirements; (5) hypertension  ≥ 99% for age (< 18 years) or ≥ 140/90 mmHg (≥ 18 years of age); (6) proteinuria ≥ 30 mg/dL or random urine protein/creatinine ratio (rUPCR) ≥ 2 mg/mg; and (7) elevated soluble terminal complement complex activity (plasma sC5b-9 above normal of < 244 ng/mL). Schoettler et al. stratified patients with any of the following poor prognostic features as high-risk TA-TMA: elevated sC5b-9, LDH ≥ 2 times the ULN, rUPCR ≥ 1 mg/mg, multiorgan dysfunction, concurrent grade II-IV acute GVHD, or infection (bacterial or viral) [67]. CNI (tacrolimus), total body irradiation (TBI), and severe acute and chronic GVHD were identified as significant risk factors correlated with a higher incidence of TA-TMA with neurological manifestations [13].

### Differential diagnosis

If CNS symptoms occur after allo-HSCT, which distinguishes it from neurological infectious (bacterial, fungal, viruses, and parasitic) complications can be crucial. Cerebrospinal fluid metagenome next-generation sequencing (mNGS) can be used for pathogen detection, which may help distinguish TA-TMA from encephalitis, progressive multifocal white matter disease (PML) caused by John Cunningham virus (JCV) infection/reactivation, and others [68]. Neuroimaging (CT-scan and/or MRI) may further help distinguish TA-TMA from cerebrovascular events, such as those hemorrhagic and ischemic in nature, malignancies, and early disease relapse. PRES is a common CNS complication of HSCT, characterized by imaging abnormalities resulting from endothelial dysfunction and often presenting with typical clinical manifestations [69]. After HSCT, several factors can contribute to PRES and are strongly associated with CNI therapy. Other contributing factors include cardiovascular risk factors, HLA mismatch, and acute GVHD [70]. The diagnosis of TA-TMA-caused PRES is based on MRI findings and the systemic diagnosis of TA-TMA with neurological complications. Chronic GVHD involving the CNS may exhibit clinical features resembling those of TA-TMA, characterized by changes in cerebrospinal fluid inflammation [71]. CNI-related CNS complications are a diagnosis of exclusion, characterized by normal neuroimaging and cerebrospinal fluid, along with concurrent use of CNI. The manifestations of CNS TMA following HSCT can overlap with various conditions, making it difficult to make a definitive diagnosis. A comprehensive evaluation is needed, considering the clinical presentation, imaging studies, and cerebrospinal fluid analysis results of each patient.

## Treatment options

### General treatment

Symptomatic treatment and complement inhibitory drugs are key for preventing irreversible organ dysfunction in TA-TMA. However, addressing the underlying cause of endothelial damage, such as GVHD or infection therapy, is fundamental for long-term management. Additional supportive measures include aggressive hypertension management and renal replacement therapy. The withdrawal or minimization of CNIs remains controversial; however, studies indicate that such actions do not improve patient survival [72]. All adjunctive therapies should be evaluated on a case-by-case basis in transplant recipients presenting with TA-TMA, and the risks and benefits should be carefully considered for each patient.

### Antibody depletion therapy

Early initiation of therapeutic plasma exchange (TPE) is thought to be beneficial in patients with TMA and multi-organ failure. By removing autoantibodies and other pathogenic factors from the plasma, TPE aims to alleviate the underlying pathology of TA-TMA. TPE can be used in patients with TA-TMA with documented Factor H autoantibodies, in conjunction with rituximab as an antibody-depletion therapy [73, 74]. Moreover, TPE can simultaneously eliminate circulating endothelial cells and factors along with the microparticles that trigger complement activation. TPE has limited efficacy in TA-TMA, particularly in the intermediate and long term. Thus, TPE should be reserved for the initial treatment of severe organ (particularly CNS) involvement but should be complemented by other measures capable of providing long-term control of TMA [75]. The anti-CD20 monoclonal antibody rituximab, which plays an antibody depletion and immune regulatory role in TA-TMA treatment, is to be administered immediately after the daily TPE procedure to achieve maximum efficacy before the next plasma exchange session. Rituximab may be effective in cases with or without TPE, or even in cases refractory to plasma exchange. In the review by Kim, 12 of 15 patients (80%) who received rituximab resulted in therapeutic efficacy [76]. In another study by Luo et al., 8 of 12 patients (66.7%) who received rituximab responded to treatment [77].

### Endothelial cell protection treatment

Defibrotide, a polydeoxyribonucleotide salt, has been shown to protect against endothelial damage by inhibiting TNFα-mediated endothelial cell apoptosis in vitro and has also demonstrated profibrinolytic, antithrombotic, anti-inflammatory, and thrombolytic activities. Defibrotide has been used to treat endothelial complications following HSCT-veno-occlusive disease (VOD), with a reported 30%–60% complete response rate [78]. In addition, defibrotide exhibits protective effects in the endothelium, potentially damaged by cyclosporine, tacrolimus, and sirolimus. Defibrotide is effective in patients with HSCT receiving prophylaxis for VOD [79]. In a study, defibrotide was previously administered as a prophylactic therapy for TA-TMA in 25 high-risk pediatric patients; in this case, the TA-TMA incidence was found to be lower than historical controls (4% vs. 18%–40%), indicating that defibrotide may reduce the risk of TA-TMA [80]. Other endothelial protectants, such as N-acetylcysteine and statins, have also been studied in the context of TA-TMA and have shown good efficacy, presenting potential therapeutic agents for TA-TMA [81, 82].

### Complement targeting drugs

**Fig. 3:**
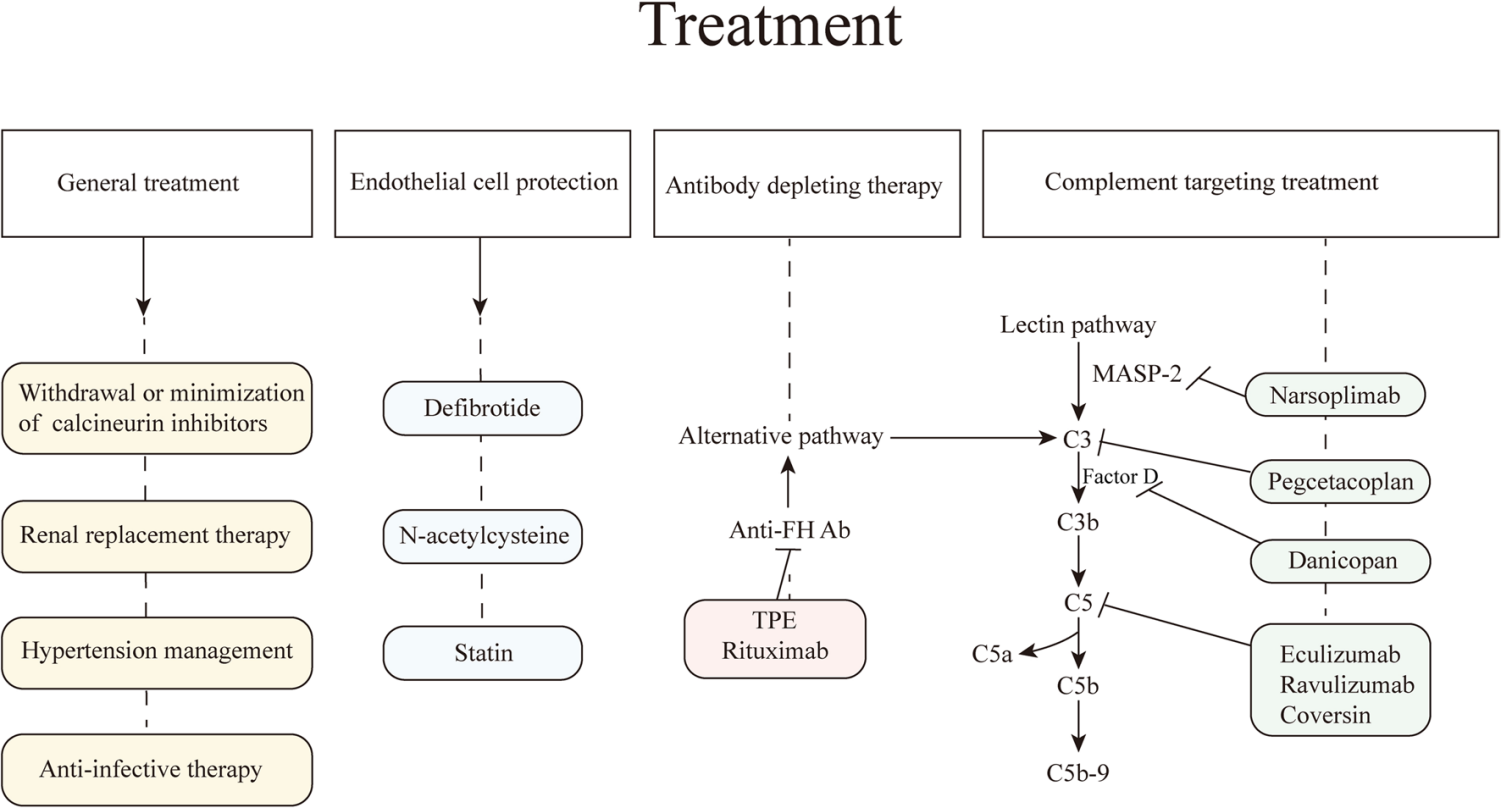
Treatment in HSCT-associated thrombotic microangiopathy. TPE, therapeutic plasma exchange

Figure 3 shows the sites of action of currently available complement inhibitory drugs. Early therapy with the C5 blocker eculizumab significantly improved outcomes in patient with high-risk TA-TMA and attenuated organ dysfunction [83]. CNS TA-TMA, a manifestation of TA-TMA, can be effectively treated with complement inhibitors.

#### C5 inhibitors

Eculizumab, a monoclonal antibody directed towards C5, prevents the formation of the C5b-9 membrane attack complex. In contrast to patients with aHUS who often receive life-long therapy with eculizumab, all patients with HSCT reported to date who responded to eculizumab were able to discontinue the therapy after TA-TMA was controlled without disease relapse [84]. A recent systematic review and meta-analysis of 116 patients from 6 studies suggested that eculizumab is safe in patients with TA-TMA and improves recovery and survival rates in both children and adults [85]. Jodele et al. described the effect of eculizumab in a prospective study comprising a subgroup of 64 out of 177 children with high-risk TA-TMA. The study demonstrated a response rate of 64% and survival rate of 66% one year after HSCT, compared with a survival rate of 16.7% in a historical untreated control cohort [66]. In a large-scale study, Mizuno et al. examined eculizumab pharmacokinetics and pharmacodynamics in 19 bleeding and 38 non-bleeding patients and, showed that in addition to bleeding, sC5b-9 and body weight were significant determinants of eculizumab clearance, in patients who needed personalized drug dosing to improve survival [84]. In some studies, HUS cases with neurological involvement and early eculizumab therapy were found to be effective, improving neurological outcomes and reducing long-term neurological sequelae [86–89]. Therefore, patients with TA-TMA exhibiting neurological manifestations should receive prophylactic or early treatment with eculizumab to optimize response and maximize the restoration of CNS function.

Ravulizumab (ALXN1210) is a new C5 inhibitor that achieves complete and sustained inhibition of complement activity with an extended dosing interval and a higher binding affinity to C5 than eculizumab [90]. The randomized controlled study (NCT04543591) assessing the effects of the long-acting C5 inhibitor ravulizumab in TA-TMA is ongoing. Danicopan is an oral complement factor D inhibitor as add-on treatment to ravulizumab or eculizumab for patients with clinically significant extravascular haemolysis [91]. Danicopan recently received approval for the treatment of patients with PNH who continue to have residual haemolytic anaemia despite treatment with a complement C5 inhibitor.

Coversin, another C5 inhibitor and a small recombinant compound, binds to C5 at a location different from that of eculizumab, and was successfully used in a patient with TA-TMA with a C5 variant that caused resistance to eculizumab treatment [92].

#### Lectin pathway inhibitor

Narsoplimab (OMS721) is a human immunoglobulin G4 monoclonal antibody that binds to and inhibits MASP-2, prevents C3 convertase formation, and blocks lectin pathway activation [93]. A phase II, single-arm, three-stage, ascending-dose-escalation study (NCT02222545) evaluated the safety and efficacy of narsoplimab in 28 adult patients with HSCT-TMA; narsoplimab treatment resulted in a high clinical response with no apparent adverse events, and the patients achieved an overall survival of 68% on day 100 [94].

#### C3 inhibitors

Pegcetacoplan is another specific complement inhibitor currently approved for PNH. Pegcetacoplan binds to complement protein C3 and its activation fragment, C3b, thereby regulating C3 cleavage and downstream activation [95]. A study to evaluate the pharmacokinetics, efficacy, safety, and tolerability of pegcetacoplan in patients with TA-TMA is currently ongoing (ClinicalTrials.gov. NCT05148299).

## Conclusions

The diagnosis of TA-TMA is associated with an unfavorable prognosis with poor outcomes and a high risk of mortality; however, it remains difficult to determine whether TA-TMA is the exact cause of death or is associated with overall clinical severity in patients with TA-TMA. Improved diagnosis of CNS TA-TMA can be achieved through increased clinical awareness, improved symptom recognition, and the use of CNS imaging as a diagnostic tool. Prospective studies are needed to better define the neurological symptoms most indicative of TA-TMA and further characterize the diagnostic imaging abnormalities in patients with TA-TMA. Novel TA-TMA biomarkers, reflecting the predisposition for injury to specific organs, need to be identified to aid earlier TA-TMA diagnosis and guide targeted therapies. Our understanding of CNS TA-TMA has advanced considerably, with some studies suggesting the role of complement-mediated endothelial injury in its pathogenesis. The endothelium represents a promising target for prophylaxis and treatment of pathologies arising from TA-TMA. As treatment strategies continue to evolve, knowledge pertaining to the key mediators of TA-TMA may assist in the diagnosis and treatment decisions.

## Data Availability

Not applicable.
